# Investigating the influence of functional electrical stimulation on motor imagery related μ-rhythm suppression

**DOI:** 10.3389/fnins.2023.1202951

**Published:** 2023-07-10

**Authors:** Lev Yakovlev, Nikolay Syrov, Alexander Kaplan

**Affiliations:** ^1^Vladimir Zelman Center for Neurobiology and Brain Rehabilitation, Skolkovo Institute of Science and Technology, Moscow, Russia; ^2^Baltic Center for Neurotechnology and Artificial Intelligence, Immanuel Kant Baltic Federal University, Kaliningrad, Russia; ^3^Laboratory for Neurophysiology and Neuro-Computer Interfaces, Lomonosov Moscow State University, Moscow, Russia

**Keywords:** motor imagery, functional electrical stimulation, sensorimotor integration, mu-rhythm suppression, sensorimotor rhythms, EEG

## Abstract

**Background:**

Motor Imagery (MI) is a well-known cognitive technique that utilizes the same neural circuits as voluntary movements. Therefore, MI practice is widely used in sport training and post-stroke rehabilitation. The suppression of the μ-rhythm in electroencephalogram (EEG) is a conventional marker of sensorimotor cortical activation during motor imagery. However, the role of somatosensory afferentation in mental imagery processes is not yet clear. In this study, we investigated the impact of functional electrical stimulation (FES) on μ-rhythm suppression during motor imagery.

**Methods:**

Thirteen healthy experienced participants were asked to imagine their right hand grasping, while a 30-channel EEG was recorded. FES was used to influence sensorimotor activation during motor imagery of the same hand.

**Results:**

We evaluated cortical activation by estimating the μ-rhythm suppression index, which was assessed in three experimental conditions: MI, MI + FES, and FES. Our findings shows that motor imagery enhanced by FES leads to a more prominent μ-rhythm suppression. Obtained results suggest a direct effect of peripheral electrical stimulation on cortical activation, especially when combined with motor imagery.

**Conclusion:**

This research sheds light on the potential benefits of integrating FES into motor imagery-based interventions to enhance cortical activation and holds promise for applications in neurorehabilitation.

## 1. Introduction

Motor imagery (MI) training is a well-known method that induces neuroplasticity and promotes motor learning (Di Rienzo et al., 2016; Ruffino et al., 2017; Ladda et al., 2021). This method has been successfully used both alone and as an additional practice for motor skill learning in athletes (Lotze and Halsband, 2006; Mizuguchi et al., 2012a), as well as to enhance the effectiveness of neurorehabilitation in patients after strokes or neurotrauma (Page et al., 2007; Carrasco and Cantalapiedra, 2016; Frolov et al., 2017). Despite a good theoretical basis and reproducible experimental and clinical results (Ramos-Murguialday et al., 2013; Pichiorri et al., 2015), there is currently no general approach to organize mental training in clinical studies, which could be the reason for numerous cases of reported low effectiveness of MI in neurorehabilitation (De Vries and Mulder, 2007). Another reason may be related to the inability of many post-stroke patients to form vivid mental images, resulting in inadequate effectiveness of MI and leading to decreased motivation and further frustration (Hougaard et al., 2022).

Brain-computer interfaces (BCIs) based on motor imagery use the specific electroencephalogram (EEG) patterns associated with the motor imagery to translate mental imagery efforts into feedback (neurofeedback). As a MI-marker BCIs utilize a μ-rhythm event related desynchronization (ERD) in human EEG, which reflects a depression in electrical oscillatory activity amplitude in the range of 8–13 Hz during cortical sensorimotor workload, such as voluntary movements, motor imagery, movement observation, tactile stimulation (Neuper et al., 2006) and tactile imagery (Yakovlev et al., 2023). Neurofeedback enables patients to control the expression of their mental efforts in each act of motor imagery (Kaplan et al., 2016; Frolov et al., 2017; Vasilyev et al., 2017; Vourvopoulos et al., 2019). Traditionally, this form of feedback is usually presented visually on a monitor screen, however in recent years, there has been a growing interest in the benefits of somatosensory neurofeedback for motor learning. Somatosensory feedback is considered more natural for motor learning approaches (Corbet et al., 2018), and may have advantages over traditional visual feedback (Cincotti et al., 2007). The somatosensory feedback could be presented in the form of orthoses (Randazzo et al., 2017), vibrotactile stimulation (Cincotti et al., 2007; Ahn et al., 2014), and functional neuromuscular stimulation (Reynolds et al., 2015; Cho et al., 2023).

It is essential to explore novel methods to modulate cortical activation levels during motor imagery, which can lead to increased motor imagery vividness and sustainability. One promising approach to enhance motor imagery involves coupling it with somatosensory stimulation (e.g., subthreshold vibrostimulation, electrical stimulation). This approach addresses the lack of somatosensory input during motor imagery (since no actual movements are being performed). It has been shown that subthreshold vibrostimulation can increase cortical activation induced by motor imagery, leading to an enhanced sensorimotor ERD (Lakshminarayanan et al., 2023a). Another well-known method successfully combined with MI is action observation (Berends et al., 2013; Vogt et al., 2013; Sun et al., 2016; Lakshminarayanan et al., 2023b). Action observation involves the mirror neuron system activation and facilitates visuomotor integration. Functional electrical stimulation (FES) of the imagined hand elicits peripheral afferent stimulation from skin receptors and muscle spindles (Reynolds et al., 2015). This approach combines the sensory modalities involved in movement execution, providing tangible functional movement that mirrors the imagined action (Kaneko et al., 2014). As a result, FES, through the combination of somatosensory stimulation and action observation, has the potential to compensate for the absence of corresponding sensory modalities during motor imagery when overt action is not feasible and can potentially be used during MI training.

One of the effective neural correlates of cortical activation is corticospinal excitability, which can be measured by single pulse transcranial magnetic stimulation (TMS; Fadiga et al., 1998). Current research has demonstrated that combining electric stimulation with motor imagery can increase corticospinal excitability up to the level of actual movement (Saito et al., 2013; Kaneko et al., 2014). In our previous study, we demonstrated that FES has a facilitating effect on corticospinal excitability that continues for a few seconds after the stimulation ends (Yakovlev et al., 2019). In this study, we examined μ-rhythm suppression as a marker of cortical activation during right-hand motor imagery coupled with FES applied to the same limb. The primary objective was to evaluate the modulatory effect of FES on μ-rhythm suppression comparing it with suppression level during FES and motor imagery alone. We hypothesized that compared to motor imagery and FES alone, FES could enhance μ-rhythm suppression during motor imagery.

## 2. Methods

### 2.1. Subjects

The study involved 13 healthy volunteers, comprising 5 women and 8 men, with a mean age of 23.4 ± 2.8 years old, all of whom had previously participated in motor imagery studies (Yakovlev et al., 2019, 2022). Each participant underwent a single experimental session that lasted for 90 min. The study protocol was approved by the ethical committee of Lomonosov Moscow State University and followed the guidelines of the Declaration of Helsinki. All participants were informed about the experimental procedures and provided their informed consent by signing a document.

### 2.2. Experimental design

The participants were seated in a comfortable armchair in the experimental room with uniform lighting. The stimuli for experimental conditions were presented on a 24-inch LCD monitor positioned in the front of the subject ~1.2 meter away from the eyes. Before each experimental session, EEG and stimulation electrodes were mounted on the participant’s scalp and hand, which took approximately 20–30 min.

**Figure 1:**
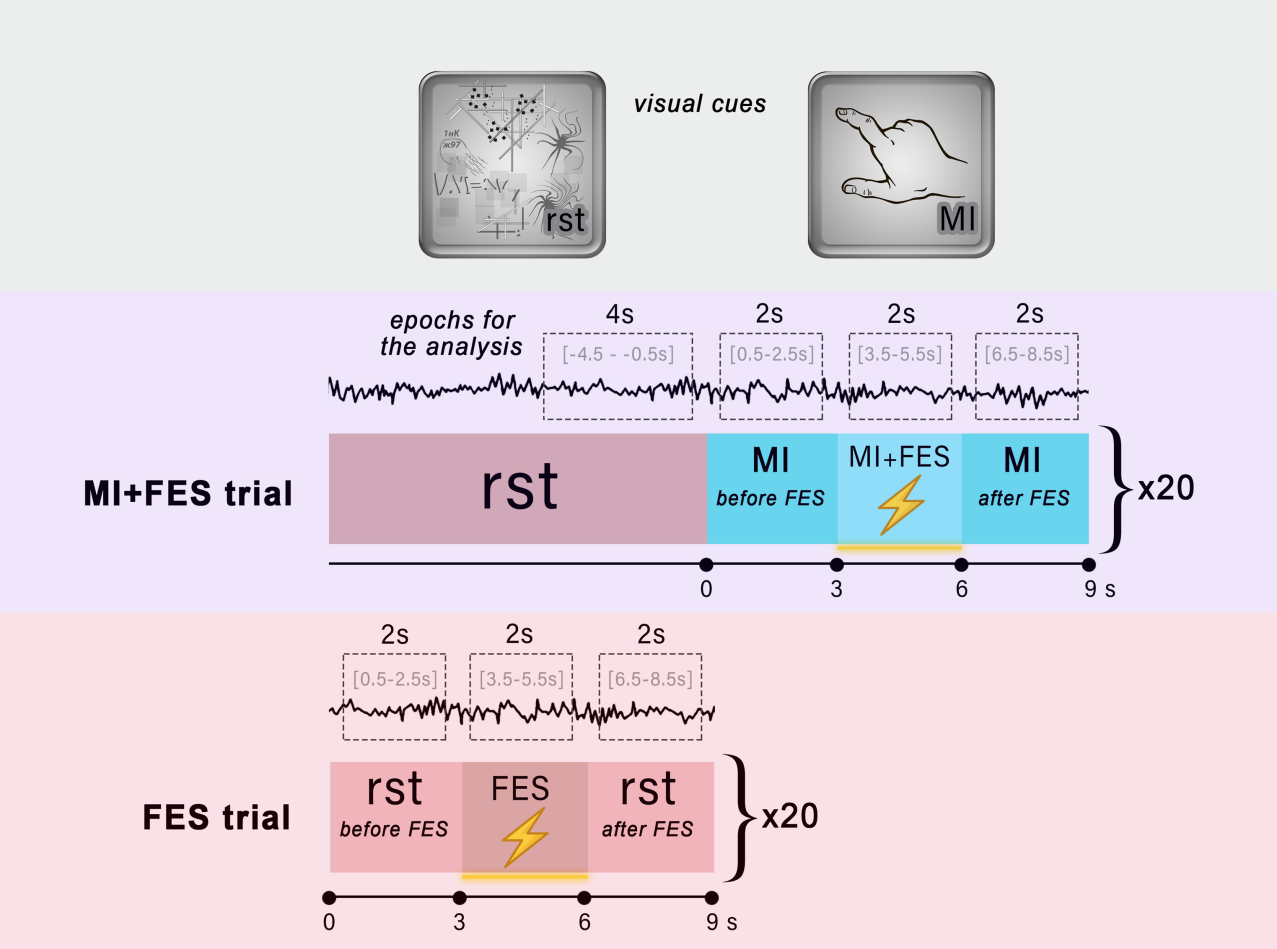
Experimental design. The top area illustrates the visual cues used in the trials to instruct participants about the task. The middle and bottom areas show the organization of the MI + FES and FES trials, respectively. The visual attention task was performed throughout the entire reference state (*rst*). The red boxes indicate the reference state, while the blue boxes represent the motor imagery task. The lightning icon symbolizes the time intervals during which functional electrical stimulation was applied. The EEG signal with limited time intervals used for epochs and analysis is shown for each condition.

During the experimental session, there were two types of trials: MI + FES trials and FES trials (Figure 1). During the MI-FES trials, participants were required to perform a kinesthetic motor imagery task involving the grasping motion of their right hand for a duration of 9 s. In the MI + FES condition, FES was applied in the *right m. flexor digitorum superficialis* (FDS) muscle area during the time interval of 3 to 6 s. As a result, the initial 3 s of the whole imagery trial were used as the MI-before condition (from 0 to 3 s), while the final 3 s were used as the MI-after condition (from 6 to 9 s). Before each MI-FES trial, a 9 s visual attention task was conducted. During visual attention task the picture with abstract graphical content (e.g., dots, lines, loops, helixes with multiple intersections) was used. In this task participants were instructed to silently count the elements on the screen at their own comfortable pace. The visual attention task served multiple purposes in this study. Firstly, on a psychological level, this task facilitated the transition from mental imagery by requiring a comparable level of attention, effectively preventing participants’ minds from wandering. Secondly, it provided a stable and reproducible cognitive state since the task was familiar to the subjects. Thirdly, the visual attention task elicited a decrease in occipital α-rhythms, while μ-rhythm exhibited an opposite response, thus effectively separating sensorimotor and visual brain networks (Vasilyev et al., 2017, 2021).

In the FES trials, participants were instructed to perform a visual attention task for a duration of 9 s. Similar to the MI + FES trials, electrical stimulation with the same parameters was initiated from 3 to 6 s during the FES trial (Figure 1). By employing these two types of trials, we were able to assess the impact of FES during motor imagery and isolate the influence of FES alone, particularly during the reference state. There were a total of 20 trials of each type, with an interstimulus interval of 150–300 ms, organized into two runs (10 trials per run). During the experimental sessions, the order of runs was randomized for each participant.

### 2.3. EEG recording

During the experimental tasks, a 30-channel monopolar EEG was recorded at 500 Hz using an NVX-52 DC amplifier (MKS, Zelenograd, Russia). Passive Ag/Cl electrodes were positioned across the sensorimotor cortex in 30 locations according to the “10/10” international system, including *F3, F4, FC5, FC3, FC1, FCz, FC2, FC4, FC6, C5, C3, C1, Cz, C2, C4, C6, CP5, CP3, CP1, CPZ, CP2, CP4, CP6, P5, P3, P1, PZ, P2, P4,* and *P6*. The reference electrode was placed at *TP10* position, and the ground electrode was placed at the *AFz* position. The skin-electrode impedance for each electrode was below 20 kΩ. The recorded signal was filtered between 0.1–75 Hz using a FIR filter, with an additional 50 Hz Notch-filter. Raw data acquisition and stimulus presentation were carried out using the BCI2000 software (Schalk et al., 2004).

### 2.4. Functional electrical stimulation

For single-channel FES, a Neuro-MVP current stimulator module (Neurosoft, Ivanovo, Russia) and a pair of 5×5 cm disposable stimulating electrodes were used. The electrodes were placed on the right-hand surface over the FDS muscle area. A single 30 μs stimulus duration was used for rhythmic electrical stimulation, resulting in functional muscle contraction (hand grasp). The stimulation pattern lasted for 3 s, with the amplitude and frequency of stimulation individually selected for each subject within the range of 36–63 mA (amplitude) and 35–60 Hz (frequency). The primary criteria for selection were functional muscle contraction and the absence of discomfort for the participants.

### 2.5. Data analysis

In this work, we assessed the μ-rhythm suppression, examined its temporal dynamics and the spatial localization. To estimate the suppression of the μ-rhythm, we applied a bandpass filter to the raw EEG signal in the range from 1 to 30 Hz using a finite impulse response (FIR) filter. The filtered signal was then spatially filtered using the Common Average Reference (CAR) spatial filter (McFarland et al., 1997). We analyzed the filtered signal in the time-frequency domain using the Wavelet Morlet transform (time window = 0.5 s; *f0* = 3 Hz central frequency; FWHM = 0.336 s). The signal was epoched corresponding to the timepoints of the explored experimental conditions: for the MI trial, the time interval was [0.5–2.5 s]; for the MI + FES trial, it was [3.5–5.5 s]; and for the FES trial, it was [3.5–5.5 s]. For the reference state, we used the time intervals of [−4.5 – −0.5 s] for the MI + FES trials, and a merged interval of [0.5–2.5 s] and [6.5–8.5 s] for the FES trials (Figure 1). Therefore, rst-epochs consisted entirely of the visual attention task performance. We excluded 0.5 s from the beginning and end of each epoch to reduce potential confounding effects related to condition transitions. Next, we normalized the time-frequency matrix values for sensorimotor trials to the reference state (*rst*, duration 4 s) and converted them into decibels to calculate the μ-rhythm suppression index for each experimental condition according to (Perry et al., 2011) (Еq. 1):


(1) Suppressionindex=10lg(PSDsmr/PSDrst)

where PSD*smr* is the averaged spectral power across the epochs of the MI, MI + FES or FES conditions in the subject-specific frequency range for each channel position. PSD*rst* defines the averaged spectral power calculated for *rst*-epochs.

To perform topographical mapping and statistical assessment, we used the sensorimotor μ-range. Data from the frequency range of 8–13 Hz were extracted for each subject and averaged across epochs and time intervals of 0.5–2.5 s after the epoch start in each channel. Statistical analysis was conducted using the Python-SciPy (Virtanen et al., 2020) and Python-MNE (Gramfort et al., 2013) modules. Given the limited sample size (*N* = 13) and the need for more robust analysis, we decided to use non-parametric statistics. We employed the non-parametric cluster-level paired *t*-test with 10 000 permutations to detect significant changes in the time-frequency dynamics of oscillatory activity and spatial distribution of the suppression index values between the experimental conditions (MI, MI + FES, FES; Maris and Oostenveld, 2007). To analyze the group data (averaged over the contralateral channels group suppression index values in the three experimental conditions), we used the Friedman test followed by Wilcoxon signed rank test for paired comparisons. We corrected the significance level using the Bonferroni method. We visualized the data using the Matplotlib graphics environment (Hunter, 2007).

## 3. Results

### 3.1. Temporal spectral dynamics

**Figure 2:**
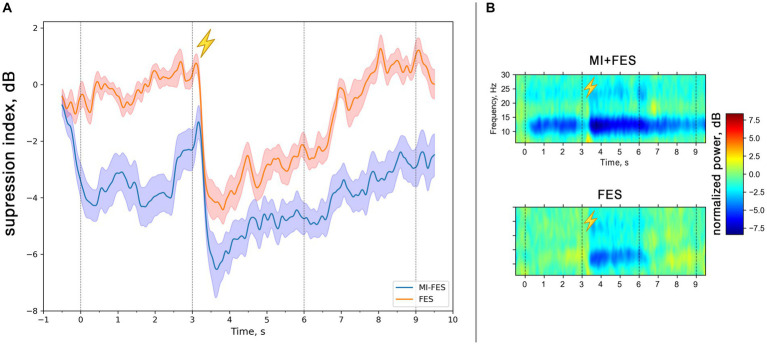
**(A)** Group averaged (*N* = 13) μ-rhythm suppression index time courses in the range 8-13 Hz for MI + FES and FES trials (*C3*-channel). The solid lines represent mean suppression index values. Color shapes correspond to standard error. The vertical lines correspond to the start and the end of the trials [0-9 s] and the start and the end of functional electrical stimulation [3-6 s]. **(B)** Group averaged (*N* = 13) time-frequency dynamics of the normalized spectral power in contralateral hemisphere (*C3*-channel) in MI + FES and FES trials. The colors indicate the suppression index (log ratio of the power in the experimental conditions over reference state). The lightning icon depicts the initiation of FES.

With the Morlet transform we obtained the time-frequency representation matrices for the motor imagery and, FES trials baselined to the resting state. Figure 2 depicts the time course of the μ-rhythm suppression with the maximum level reached after 600 ms after the stimulation onset (Figure 2A). It should be noted that in the MI + FES trials, electrical stimulation was initiated during the motor imagery condition, while in the FES trials, the hand was stimulated during the reference state. This had an impact on the time courses, where in the case of MI-FES trials, the suppression (Figure 2A, blue line) remained present before and after stimulation, whereas in the FES trials, it started and returned to near-zero levels (Figure 2B, red line). Figure 2B shows the group-average temporal spectral dynamics for the trials (shown for *C3*-channel): there is an μ-rhythm suppression in the range of 8–13 Hz manifested both during MI + FES and FES trials and visible β-suppression in the range of 20–26 Hz. Comparing these trials by the non-parametric cluster-level paired *t*-test has shown statistical signifiable differences in only in μ-band (*p* < 0.001) but not for β-band. In connection with this, further analysis was conducted for the μ-rhythm only.

### 3.2. The topographic localization of μ-rhythm suppression

**Figure 3:**
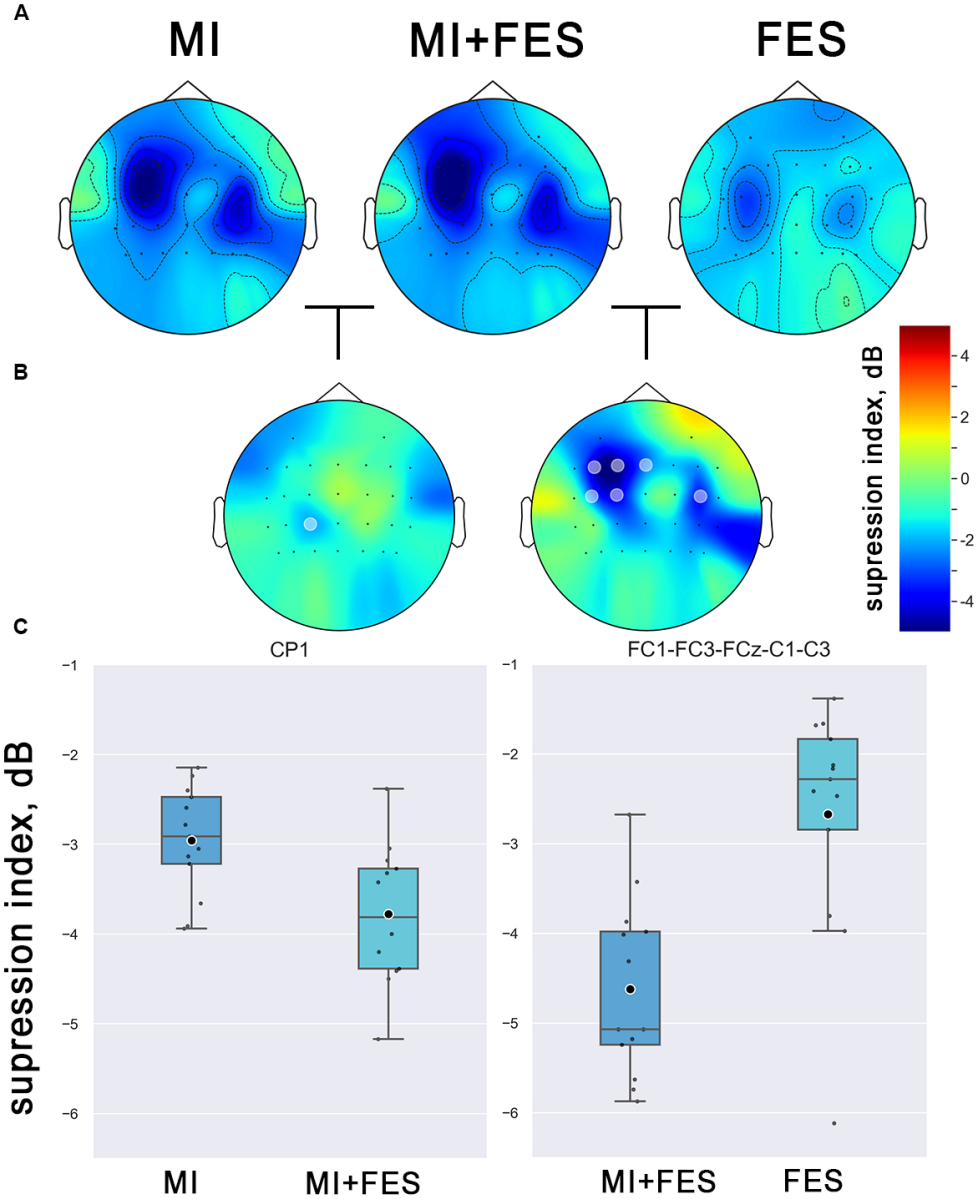
**(A)** Group averaged (*N* = 13) topographical representations of the μ-rhythm suppression index values (8–13 Hz) in explored conditions. The colors indicate the suppression index (log ratio of the power in the experimental conditions over reference state). **(B)** Topographical representation of the differences between conditions (marked channels with statistical significance *p* < 0.01, non-parametric cluster permutation *t*-test). **(С)** Contralateral μ-suppression index values in the obtained clusters of channels with statistical significance (channels, marked on Figure 4B).

To determine the topographical localization of the sensorimotor response, the μ-rhythm suppression index values for each electrode position across all experimental conditions were compared using the nonparametric permutation *t*-test. The resulting group-averaged (*N* = 13) topographic distribution patterns for μ-suppresion index for all experimental conditions are presented in Figure 3A.

The μ-rhythm suppression induced by motor imagery and FES was observed to be more prominent in the central EEG channels over the sensorimotor cortical areas, with contralateral dominance. Notably, a significantly stronger μ-suppression was observed in the MI + FES condition compared to MI and FES alone (as shown in Figure 3B). Significant differences (*p* < 0.01) were obtained for the group of *FC1-FC3-FCz-C1-C3-C4* channels between FES and MI + FES conditions (however, for further paired comparisons, we discarded the *C4* channel to avoid contaminating data from the central-contralateral group with data from the ipsilateral channel). For the MI + FES vs. MI comparison, there was a statistically significant difference in only the *CP1* channel (the exact statistical parameters are also listed in Table 1).

**Table 1:** The results of performed non-parametric cluster permutation *t*-test.

Channel	*t-*stat	*p*-value	Comparison
*FC1*	−4.547	0.001	MI + FES vs. FES
*FC3*	−5.091	0.006	MI + FES vs. FES
*FCz*	−6.063	0.006	MI + FES vs. FES
*C1*	−5.972	0.001	MI + FES vs. FES
*C3*	−7.265	0.006	MI + FES vs. FES
*C4*	−5.078	0.003	MI + FES vs. FES
*CP1*	−4.854	0.003	MI + FES vs. MI

### 3.3. μ-rhythm suppression during motor imagery coupled with FES

The present study aimed to investigate the modulation of μ-rhythm suppression index values under different experimental conditions. To this end, we conducted a statistical analysis using a Friedman’s test with post-hoc analysis for the group of contralateral channels (*FC1-FC3-FCz-C1-C3-CP1*) that was selected based on permutation-based clusters (see Figure 3).

**Figure 4:**
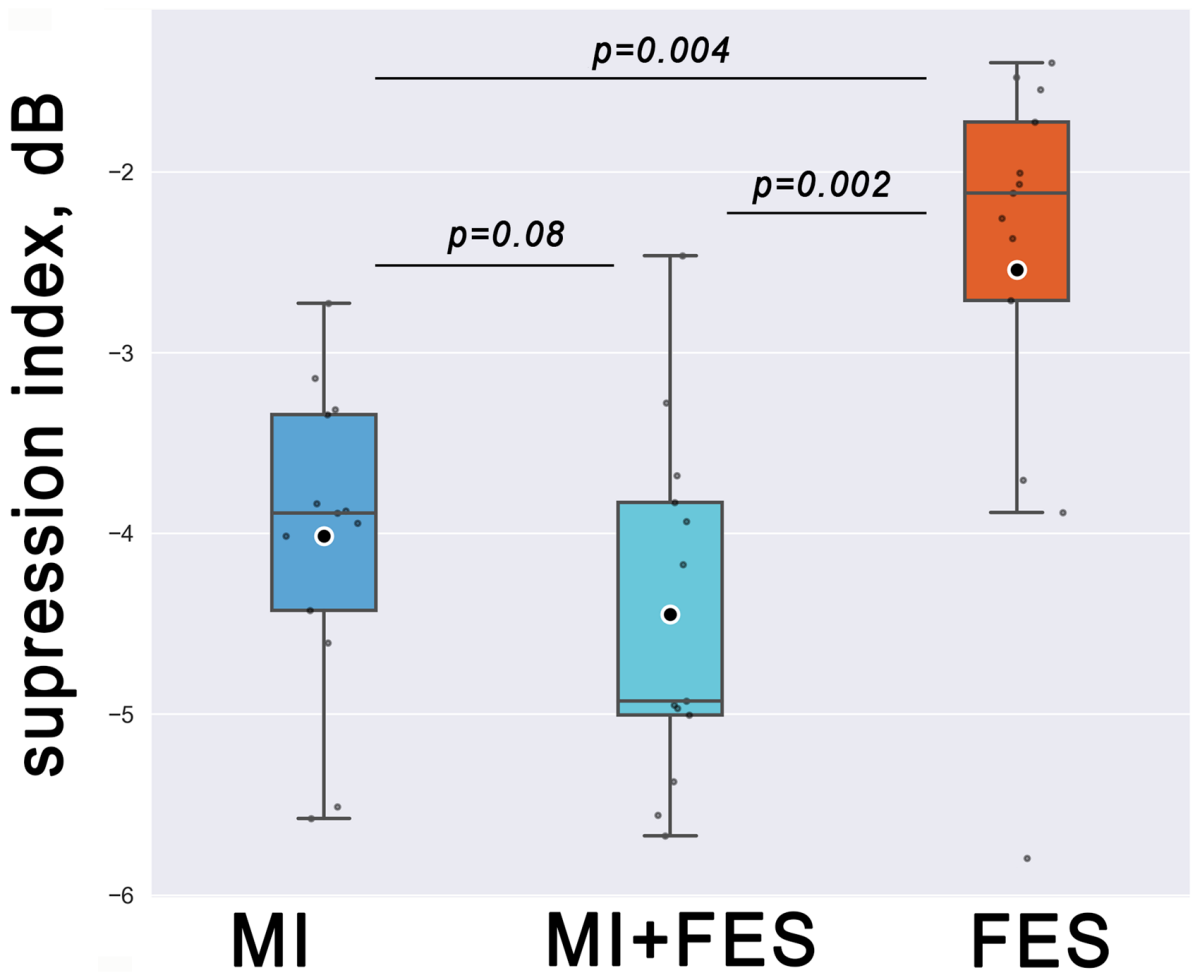
Group level (*N* = 13) μ-suppression index values in the group of *FC1-FC3-FCz-C1-C3-CP1* channels for explored somatosensory conditions. Horizontal lines within the boxes correspond to the median values, boxes - to the interquartile range (IQR) and [Q1-1.5*IQR; Q3 + 1.5*IQR] range is shown by whiskers. Circles represent mean values for the group. Corrected *p*-values are shown for the Wilcoxon signed-rank test with Bonferroni correction.

The Friedman test revealed significant differences in μ-suppression index values, which were used as dependent variables, among the experimental conditions (MI, MI + FES, FES) as independent variables (*χ*^2^ = 15.846; *p* < 0.001). The most prominent μ-suppression was reached during MI + FES condition, which was higher than both MI and FES alone. However, paired tests showed statistical significance only for the MI + FES vs. FES comparison (*p* = 0.002). For the comparison of MI + FES vs. MI conditions, the initial significant differences were eliminated after the Bonferroni correction (corrected *p* = 0.08; uncorrected *p*-value 0.027). Paired comparisons between μ-suppression index values in the FES and MI conditions showed a significant difference (*p* = 0.004; see Figure 4).

## 4. Discussion

We have obtained μ-rhythm suppression in the contralateral group of channels during motor imagery, enhanced by FES. At the same time, μ-suppression level in each of the conditions separately was lower. It is well known that FES, as well as motor imagery, lead to desynchronization of EEG sensorimotor rhythms (Pfurtscheller and Da Silva, 1999; Pfurtscheller et al., 2003). Studies that investigate the joint influence of FES and motor imagery on cortical sensorimotor activation have mainly focused on classification accuracy and stability in BCI performance parameters. Previous studies have shown that the use of FES in MI-based BCI enhances classification accuracy and stability in BCI performance (Pfurtscheller et al., 2003; Reynolds et al., 2015; Bhattacharyya et al., 2016; Ren et al., 2020). The aim of this study was to investigate a more fundamental aspect of the effect of FES on sensorimotor activation independent of the BCI task.

According to the commonly accepted view, μ-rhythm suppression is one of the cortical activation markers manifested in a number of sensorimotor tasks, including motor imagery (Pfurtscheller and Neuper, 1997). The μ-rhythm suppression level reflects the size of the neuronal ensemble involved in task execution, which is confirmed by studies examining the performance of tasks with varying degrees of complexity (Pineda, 2005). However, the presence or absence of suppressed μ-rhythm may not directly correspond to the intensity and quality of motor imagery. In several studies, there has been a demonstrated lack of correlation between two common correlates of cortical activation – suppression of the μ-rhythm in EEG and corticospinal excitability measured by single-pulse TMS (Kaplan et al., 2016; Vasilyev et al., 2017). Nevertheless, for technologies based on brain-computer interaction, motor imagery induced μ-rhythm suppression is used as one of the main classification features that allows for feedback system control (Kaplan et al., 2016). Weak or absent μ-suppression is one of the causes of BCI illiteracy, which limits the ability of participants to use BCIs. This limitation impedes the use of BCI technology, including the development of rehabilitation training systems (Allison and Neuper, 2010; Blankertz et al., 2010; Vidaurre and Blankertz, 2010).

Normally, movement execution is accompanied by intense multisensory integration, in which the most important modalities are proprioceptive and visual (Stevens, 2005). During the motor imagery, the sensory feedback corresponding to the movement is absent. According to the emulation theory (Grush, 2004) and the results of recent studies, the inverse afferentation significantly influences the processes of forming mental images related to movements (Naito et al., 2002; Mizuguchi et al., 2009, 2012b). In the work (Hanakawa et al., 2008), the authors concluded that the process of a mental motor act can be activated by sensory stimulation or by voluntary reference to so-called “motoric memory.” It follows that providing congruent somatosensory afferentation through the stimulation of motor and sensory nerves of the limb can facilitate the perception of one’s own limb and improve the motor imagery quality. Evidence in favor of this hypothesis has been obtained from studies using various types of neuromuscular stimulation (Reynolds et al., 2015; Corbet et al., 2018). In particular, the use of subthreshold neuromuscular stimulation combined with movement representation enhanced desynchronization of sensorimotor rhythms in EEG (Corbet et al., 2018). In another study, Reynolds and colleagues showed that FES combined with motor imagery leads to an increase in ERD (Reynolds et al., 2015). Despite the similarity to the study by Reynolds et al. there are significant differences between the mentioned study and the present work. Firstly, they used FES as feedback for EMG control – that is, the initiation of stimulation depended on the ERD level in each trial. In the present work, stimulation was applied regardless of the μ-rhythm suppression level. Secondly, the differences lie in the methods used and experimental design: in the present work, multichannel EEG was used for the topographical mapping of suppression index. As noted earlier, in our work, FES was used outside the BCI control loop and was applied automatically during motor imagery trials. Nevertheless, the data obtained in our work do not contradict and complement the already obtained information about the potentiating effect of FES on the sensorimotor μ-rhythm dynamics in EEG.

We observed an increase of the μ-rhythm suppression induced by FES during motor imagery comparing to the MI and FES alone. This increase could be explained by the amplification of the internal mental process of motor imagery due to the additional sensory afferentation resulting from transcutaneous electrical stimulation. The afferentation was not limited to somatosensory and proprioceptive modalities, but also had a visual component - participants could see their hand contracting during motor imagery combined with FES. Since it is known that internal focus of attention is one of the key factors influencing the effects of movement imagery (Sakurada et al., 2016), it can be assumed that FES allows for the most effective concentration on the emerging sensations in the limb. The presence of congruent sensory stimulation corresponding to the imagined movement can facilitate the perception of one’s own limb. Proprioception activated by FES leads to full muscle contraction, resulting in a deeper kinesthetic experience for the individual. The resulting sensations can be used to support motor imagery (Ren et al., 2020). Taking this logic into account, similar effects can be expected not only at the level of the μ-rhythm suppression but also on corticospinal excitability. Data on the positive impact of peripheral stimulation on excitability have been confirmed in several studies (Saito et al., 2013; Kaneko et al., 2014; Yakovlev et al., 2019).

Although our study yielded promising results, it is important to mention several limitations that must be taken into account. First, the sample size of our study was relatively small, with only 13 participants. As a result, the generalizability of our findings may be limited.

Another limitation of our study is its focus on short-term effects immediately after the motor imagery combined with FES application. The long-term effects and durability of the observed improvements in motor imagery vividness and sustainability remain unknown. To assess the lasting benefits of the proposed approach, future studies should examine the persistence of these effects over time. Given the vividness, it would greatly improve future studies to include surveys to assess how perceptual experiences caused by FES affect subjective features of the kinesthetic image.

Furthermore, our study specifically used right-handed motor imagery tasks in combination with FES. It is uncertain whether the observed effects can be generalized to other types of motor imagery tasks or different body parts. To gain a deeper understanding of the transferability of our findings, further research is warranted to examine a broader range of motor tasks and body regions.

## 5. Conclusion

We obtained evidence of μ-suppression potentiation during motor imagery using functional electrical stimulation. The present study contributes to the growing body of literature on the neural mechanisms underlying motor control and rehabilitation and may have implications for the development of novel interventions to improve motor function in individuals with neurological disorders including BCI applications. More detailed research in this area may shed light on the emergence of neuroplasticity mechanisms in post-stroke motor rehabilitation.

## Data availability statement

The raw data supporting the conclusions of this article will be made available by the authors, without undue reservation.

## Ethics statement

The studies involving human participants were reviewed and approved by the ethical committee of Lomonosov Moscow State University. The participants provided their written informed consent to participate in this study.

## Author contributions

AK conceived of the presented idea and supervised the project. LY and NS carried out the experimental session. LY processed the data. All authors discussed the results, approved the submitted version and contributed to the final version of the manuscript.

## Funding

The work was supported by the Russian Science Foundation, grant No. 21-75-30024.

## Conflict of interest

The authors declare that the research was conducted in the absence of any commercial or financial relationships that could be construed as a potential conflict of interest.

## Publisher’s note

All claims expressed in this article are solely those of the authors and do not necessarily represent those of their affiliated organizations, or those of the publisher, the editors and the reviewers. Any product that may be evaluated in this article, or claim that may be made by its manufacturer, is not guaranteed or endorsed by the publisher.

## References

[ref1] Ahn S. Ahn M. Cho H. Chan Jun S. (2014). Achieving a hybrid brain–computer interface with tactile selective attention and motor imagery. J. Neural Eng. 11:066004. doi: 10.1088/1741-2560/11/6/066004, PMID: 25307730

[ref2] Allison B. Z. Neuper C. (2010). Could anyone use a BCI?. Brain-computer interfaces: Applying our minds to human-computer interaction, 35–54.

[ref4] Berends H. I. Wolkorte R. Ijzerman M. J. Van Putten M. J. A. M. (2013). Differential cortical activation during observation and observation-and-imagination. Exp. Brain Res. 229, 337–345. doi: 10.1007/s00221-013-3571-8, PMID: 23771606

[ref5] Bhattacharyya S. Clerc M. Hayashibe M. (2016). A study on the effect of Electrical Stimulation during motor imagery learning in Brain-computer interfacing. In 2016 ieee international conference on systems, man, and cybernetics (smc). pp. 002840–002845. IEEE.

[ref6] Blankertz B. Sannelli C. Halder S. Hammer E. M. Kübler A. Müller K. R.. (2010). Neurophysiological predictor of SMR-based BCI performance. NeuroImage 51, 1303–1309. doi: 10.1016/j.neuroimage.2010.03.022, PMID: 20303409

[ref7] Carrasco D. G. Cantalapiedra J. A. (2016). Effectiveness of motor imagery or mental practice in functional recovery after stroke: a systematic review. Neurología 31, 43–52. doi: 10.1016/j.nrleng.2013.02.008 23601759

[ref8] Cho W. Vidaurre C. An J. Birbaumer N. Ramos-Murguialday A. (2023). Cortical processing during robot and functional electrical stimulation. Front. Syst. Neurosci. 17:1045396. doi: 10.3389/fnsys.2023.1045396, PMID: 37025164 PMC10070684

[ref9] Cincotti F. Kauhanen L. Aloise F. Palomäki T. Caporusso N. Jylänki P.. (2007). Vibrotactile feedback for brain-computer interface operation. Comput. Intell. Neurosci. 2007, 1–12. doi: 10.1155/2007/48937, PMID: 18354734 PMC2267023

[ref10] Corbet T. Iturrate I. Pereira M. Perdikis S. Millán J. D. R. (2018). Sensory threshold neuromuscular electrical stimulation fosters motor imagery performance. NeuroImage 176, 268–276. doi: 10.1016/j.neuroimage.2018.04.005, PMID: 29689307

[ref11] De Vries S. Mulder T. (2007). Motor imagery and stroke rehabilitation: a critical. J. Rehabil. Med. 39, 5–13. doi: 10.2340/16501977-0020, PMID: 17225031

[ref12] Di Rienzo F. Debarnot U. Daligault S. Saruco E. Delpuech C. Doyon J.. (2016). Online and offline performance gains following motor imagery practice: a comprehensive review of behavioral and neuroimaging studies. Front. Hum. Neurosci. 10:315. doi: 10.3389/fnhum.2016.00315 27445755 PMC4923126

[ref13] Fadiga L. Buccino G. Craighero L. Fogassi L. Gallese V. Pavesi G. (1998). Corticospinal excitability is specifically modulated by motor imagery: a magnetic stimulation study. Neuropsychologia 37, 147–158. doi: 10.1016/S0028-3932(98)00089-X, PMID: 10080372

[ref14] Frolov A. A. Mokienko O. Lyukmanov R. Biryukova E. Kotov S. Turbina L.. (2017). Post-stroke rehabilitation training with a motor-imagery-based brain-computer interface (BCI)-controlled hand exoskeleton: a randomized controlled multicenter trial. Front. Neurosci. 11:400. doi: 10.3389/fnins.2017.00400, PMID: 28775677 PMC5517482

[ref15] Gramfort A. Luessi M. Larson E. Engemann D. A. Strohmeier D. Brodbeck C.. (2013). MEG and EEG data analysis with MNE-Python. Front. Neurosci. 7:267. doi: 10.3389/fnins.2013.00267 24431986 PMC3872725

[ref01] Grush R. (2004). The emulation theory of representation: Motor control, imagery, and perception. Behav. Brain Sci. 27, 377–396. 15736871 10.1017/s0140525x04000093

[ref02] Hanakawa T. Dimyan M. A. Hallett M. (2008). Motor planning, imagery, and execution in the distributed motor network: a time-course study with functional MRI. Cerebral Cortex 18, 2775–2788. 18359777 10.1093/cercor/bhn036 PMC2583155

[ref16] Hougaard B. I. Knoche H. Kristensen M. S. Jochumsen M. (2022). Modulating frustration and agency using fabricated input for motor imagery BCIs in stroke rehabilitation. IEEE Access 10, 72312–72327. doi: 10.1109/ACCESS.2022.3188103

[ref17] Hunter J. D. (2007). Matplotlib: a 2D graphics environment. Comp. Sci. Eng. 9, 90–95. doi: 10.1109/MCSE.2007.55

[ref18] Kaneko F. Hayami T. Aoyama T. Kizuka T. (2014). Motor imagery and electrical stimulation reproduce corticospinal excitability at levels similar to voluntary muscle contraction. J. Neuroeng. Rehabil. 11, 94–97. doi: 10.1186/1743-0003-11-94 24902891 PMC4113028

[ref19] Kaplan A. Vasilyev A. Liburkina S. Yakovlev L. (2016). “Poor BCI performers still could benefit from motor imagery training.” in *Foundations of Augmented Cognition: Neuroergonomics and Operational Neuroscience: 10th International Conference, AC 2016, Held as Part of HCI International 2016, Toronto, ON, Canada, July 17–22, 2016, Proceedings, Part I 10*. pp. 46–56. Springer International Publishing.

[ref20] Ladda A. M. Lebon F. Lotze M. (2021). Using motor imagery practice for improving motor performance–a review. Brain Cogn. 150:105705. doi: 10.1016/j.bandc.2021.105705, PMID: 33652364

[ref21] Lakshminarayanan K. Shah R. Daulat S. Moodley V. Yao Y. Madathil D. (2023a). The effect of combining action observation in virtual reality with Kinesthetic motor imagery on cortical activity. Front. Neurosci. 17:1021. doi: 10.3389/fnins.2023.1201865 PMC10299830 37383098

[ref22] Lakshminarayanan K. Shah R. Yao Y. Madathil D. (2023b). The effects of subthreshold vibratory noise on cortical activity during motor imagery. Mot. Control. 1, 1–14. doi: 10.1123/mc.2022-0061 36801814

[ref23] Lotze M. Halsband U. (2006). Motor imagery. J. Physiol. 99, 386–395. doi: 10.1016/j.jphysparis.2006.03.012 16716573

[ref24] Maris E. Oostenveld R. (2007). Nonparametric statistical testing of EEG-and MEG-data. J. Neurosci. Methods 164, 177–190. doi: 10.1016/j.jneumeth.2007.03.024, PMID: 17517438

[ref25] McFarland D. J. McCane L. M. David S. V. Wolpaw J. R. (1997). Spatial filter selection for EEG-based communication. Electroencephalogr. Clin. Neurophysiol. 103, 386–394. doi: 10.1016/S0013-4694(97)00022-2, PMID: 9305287

[ref26] Mizuguchi N. Nakata H. Uchida Y. Kanosue K. (2012a). Motor imagery and sport performance. J. Phys. Fitness Sports Med. 1, 103–111. doi: 10.7600/jpfsm.1.103

[ref27] Mizuguchi N. Sakamoto M. Muraoka T. Kanosue K. (2009). Influence of touching an object on corticospinal excitability during motor imagery. Exp. Brain Res. 196, 529–535. doi: 10.1007/s00221-009-1875-5 19504259

[ref28] Mizuguchi N. Sakamoto M. Muraoka T. Moriyama N. Nakagawa K. Nakata H.. (2012b). Influence of somatosensory input on corticospinal excitability during motor imagery. Neurosci. Lett. 514, 127–130. doi: 10.1016/j.neulet.2012.02.073, PMID: 22402190

[ref29] Naito E. Kochiyama T. Kitada R. Nakamura S. Matsumura M. Yonekura Y.. (2002). Internally simulated movement sensations during motor imagery activate cortical motor areas and the cerebellum. J. Neurosci. 22, 3683–3691. doi: 10.1523/JNEUROSCI.22-09-03683.2002, PMID: 11978844 PMC6758350

[ref30] Neuper C. Wörtz M. Pfurtscheller G. (2006). ERD/ERS patterns reflecting sensorimotor activation and deactivation. Prog. Brain Res. 159, 211–222. doi: 10.1016/S0079-6123(06)59014-4, PMID: 17071233

[ref31] Page S. J. Levine P. Leonard A. (2007). Mental practice in chronic stroke: results of a randomized, placebo-controlled trial. Stroke 38, 1293–1297. doi: 10.1161/01.STR.0000260205.67348.2b 17332444

[ref32] Perry A. Stein L. Bentin S. (2011). Motor and attentional mechanisms involved in social interaction—evidence from mu and alpha EEG suppression. NeuroImage 58, 895–904. doi: 10.1016/j.neuroimage.2011.06.060, PMID: 21742042

[ref33] Pfurtscheller G. Da Silva F. L. (1999). Event-related EEG/MEG synchronization and desynchronization: basic principles. Clin. Neurophysiol. 110, 1842–1857. doi: 10.1016/S1388-2457(99)00141-8, PMID: 10576479

[ref34] Pfurtscheller G. Müller G. R. Pfurtscheller J. Gerner H. J. Rupp R. (2003). ‘Thought’–control of functional electrical stimulation to restore hand grasp in a patient with tetraplegia. Neurosci. Lett. 351, 33–36. doi: 10.1016/S0304-3940(03)00947-9, PMID: 14550907

[ref35] Pfurtscheller G. Neuper C. (1997). Motor imagery activates primary sensorimotor area in humans. Neurosci. Lett. 239, 65–68. doi: 10.1016/S0304-3940(97)00889-6 9469657

[ref36] Pichiorri F. Morone G. Petti M. Toppi J. Pisotta I. Molinari M.. (2015). Brain–computer interface boosts motor imagery practice during stroke recovery. Ann. Neurol. 77, 851–865. doi: 10.1002/ana.24390, PMID: 25712802

[ref37] Pineda J. A. (2005). The functional significance of mu rhythms: translating “seeing” and “hearing” into “doing”. Brain Res. Rev. 50, 57–68. doi: 10.1016/j.brainresrev.2005.04.005, PMID: 15925412

[ref38] Ramos-Murguialday A. Broetz D. Rea M. Läer L. Yilmaz Ö. Brasil F. L.. (2013). Brain–machine interface in chronic stroke rehabilitation: a controlled study. Ann. Neurol. 74, 100–108. doi: 10.1002/ana.23879, PMID: 23494615 PMC3700597

[ref39] Randazzo L. Iturrate I. Perdikis S. Millán J. D. R. (2017). Mano: a wearable hand exoskeleton for activities of daily living and neurorehabilitation. IEEE Robot. Autom. Lett. 3, 500–507. doi: 10.1109/LRA.2017.2771329

[ref40] Ren S. Wang W. Hou Z. G. Liang X. Wang J. Shi W. (2020). Enhanced motor imagery based brain-computer interface via FES and VR for lower limbs. IEEE Trans. Neural Syst. Rehabil. Eng. 28, 1846–1855. doi: 10.1109/TNSRE.2020.3001990, PMID: 32746291

[ref41] Reynolds C. Osuagwu B. A. Vuckovic A. (2015). Influence of motor imagination on cortical activation during functional electrical stimulation. Clin. Neurophysiol. 126, 1360–1369. doi: 10.1016/j.clinph.2014.10.007, PMID: 25454278 PMC4493293

[ref42] Ruffino C. Papaxanthis C. Lebon F. (2017). Neural plasticity during motor learning with motor imagery practice: review and perspectives. Neuroscience 341, 61–78. doi: 10.1016/j.neuroscience.2016.11.023, PMID: 27890831

[ref43] Saito K. Yamaguchi T. Yoshida N. Tanabe S. Kondo K. Sugawara K. (2013). Combined effect of motor imagery and peripheral nerve electrical stimulation on the motor cortex. Exp. Brain Res. 227, 333–342. doi: 10.1007/s00221-013-3513-5, PMID: 23591692

[ref44] Sakurada T. Hirai M. Watanabe E. (2016). Optimization of a motor learning attention-directing strategy based on an individual’s motor imagery ability. Exp. Brain Res. 234, 301–311. doi: 10.1007/s00221-015-4464-9, PMID: 26466828

[ref45] Schalk G. McFarland D. J. Hinterberger T. Birbaumer N. Wolpaw J. R. (2004). BCI2000: a general-purpose brain-computer interface (BCI) system. IEEE Trans. Biomed. Eng. 51, 1034–1043. doi: 10.1109/TBME.2004.827072, PMID: 15188875

[ref46] Stevens J. A. (2005). Interference effects demonstrate distinct roles for visual and motor imagery during the mental representation of human action. Cognition 95, 329–350. doi: 10.1016/j.cognition.2004.02.008, PMID: 15788162

[ref47] Sun Y. Wei W. Luo Z. Gan H. Hu X. (2016). Improving motor imagery practice with synchronous action observation in stroke patients. Top. Stroke Rehabil. 23, 245–253. doi: 10.1080/10749357.2016.1141472, PMID: 27077982

[ref48] Vasilyev A. Liburkina S. Yakovlev L. Perepelkina O. Kaplan A. (2017). Assessing motor imagery in brain-computer interface training: psychological and neurophysiological correlates. Neuropsychologia 97, 56–65. doi: 10.1016/j.neuropsychologia.2017.02.005, PMID: 28167121

[ref49] Vasilyev A. N. Nuzhdin Y. O. Kaplan A. Y. (2021). Does real-time feedback affect sensorimotor eeg patterns in routine motor imagery practice? Brain Sci. 11:1234. doi: 10.3390/brainsci11091234, PMID: 34573253 PMC8469546

[ref50] Vidaurre C. Blankertz B. (2010). Towards a cure for BCI illiteracy. Brain Topogr. 23, 194–198. doi: 10.1007/s10548-009-0121-6, PMID: 19946737 PMC2874052

[ref51] Virtanen P. Gommers R. Oliphant T. E. Haberland M. Reddy T. Cournapeau D.. (2020). SciPy 1.0: fundamental algorithms for scientific computing in Python. Nat. Methods 17, 261–272. doi: 10.1038/s41592-019-0686-2, PMID: 32015543 PMC7056644

[ref52] Vogt S. Di Rienzo F. Collet C. Collins A. Guillot A. (2013). Multiple roles of motor imagery during action observation. Front. Hum. Neurosci. 7:807. doi: 10.3389/fnhum.2013.00807 24324428 PMC3839009

[ref53] Vourvopoulos A. Pardo O. M. Lefebvre S. Neureither M. Saldana D. Jahng E.. (2019). Effects of a brain-computer interface with virtual reality (VR) neurofeedback: a pilot study in chronic stroke patients. Front. Hum. Neurosci. 13:210. doi: 10.3389/fnhum.2019.00210 31275126 PMC6593205

[ref03] Yakovlev L. Kuznetsov I. Syrov N. Kaplan A. (2022). “Motor Imagery Training Improves Reaction Time in Mouse Aiming Task,” in Human Interaction, Emerging Technologies and Future Systems V: Proceedings of the 5th International Virtual Conference on Human Interaction and Emerging Technologies, IHIET 2021, August 27-29, 2021 and the 6th IHIET: Future Systems (IHIET-FS 2021), October 28-30, 2021, France. (Springer International Publishing), 1063–1068.

[ref54] Yakovlev L. Syrov N. Miroshnikov A. Lebedev M. Kaplan A. (2023). Event-related desynchronization induced by tactile imagery: An EEG study. eNeuro 10, ENEURO.0455–ENEU22.2023. doi: 10.1523/ENEURO.0455-22.2023, PMID: 37263791 PMC10275400

[ref55] Yakovlev L. V. Syrov N. V. Morozova E. Y. Kaplan A. Y. (2019). Corticospinal excitability in humans during motor imagery coupled with functional electrical stimulation. Mosc. Univ. Biol. Sci. Bull. 74, 183–187. doi: 10.3103/S0096392519030118

